# HSP90 inhibition disrupts telomere maintenance and promotes chromosomal instability (CIN) in cancer cells

**DOI:** 10.21203/rs.3.rs-8369668/v1

**Published:** 2026-02-11

**Authors:** Hee-Sheung Lee, Jung-Hyun Kim, Mikhail Liskovykh, Neckers Len, Natalay Kouprina, Vladimir Larionov

**Affiliations:** National Cancer Institute; National Cancer Institute; National Cancer Institute; National Cancer Institute; National Cancer Institute; National Cancer Institute

**Keywords:** HSP90 inhibitors, chromosomal instability, CIN, telomere dysfunction, human artificial chromosome, HAC

## Abstract

**Background:**

Heat shock protein 90 (HSP90) stabilizes numerous oncogenic client proteins, including factors required for telomere maintenance. Telomere dysfunction triggers chromosomal instability (CIN). To quantify telomere-directed activity and separate it from general mitotic effects, we utilized a human artificial chromosome (HAC) assay with isogenic lines carrying a linear, telomere-containing EGFP HAC or a circular, telomere-lacking EGFP HAC.

**Methods:**

Four HSP90 inhibitors (TAS-116, XL-888, SNX-2112, 17-AAG) were tested at each compound’s cell-specific LC50 in HT1080 (linear and circular HACs) and HEK293 (linear HAC) cells, with GRN163L as a positive control. HAC loss was quantified by flow cytometry. Telomere length was measured by Southern blot and qPCR in parental HT1080 and HEK293 cells, and telomere signal intensity by FISH in HEK293. Telomere dysfunction (TIFs; γH2AX/TRF2) and micronuclei (MNi) were also scored.

**Results:**

TAS-116 showed the strongest telomere-specific activity among HSP90 inhibitors, significantly increasing linear HAC loss in both HT1080 and HEK293 while circular HACs showed only minimal instability. TAS-116 shortened telomeres in both lines (qPCR: ~1.8-fold in HT1080; ~2-fold in HEK293) and, in HEK293, reduced FISH telomere signal to 59.66% of control. TAS-116 also increased telomere-associated damage (~ 1.8 TIFs per TIF-positive nucleus), DNA double-strand breaks, and micronuclei relative to the other HSP90 inhibitors.

**Conclusions:**

TAS-116 consistently disrupts telomere maintenance—driving linear HAC loss, telomere shortening, reduced FISH signal, elevated TIFs, and increased MNi—thereby validating the HAC-based framework for discriminating telomere-directed activity. These findings support the therapeutic promise of HSP90 inhibition against telomere maintenance in cancer.

## Introduction

Telomeres, repetitive TTAGGG sequences at chromosome ends, preserve genomic integrity by preventing DNA damage responses and aberrant repair events such as chromosome fusions [1]. In most somatic cells, telomeres progressively shorten with each division due to the end-replication problem. In contrast, 85–90% of cancers reactivate telomerase, a ribonucleoprotein complex comprising the catalytic subunit hTERT and the RNA template hTR, to sustain telomere length and enable unlimited proliferation [2]. Because telomerase activity is nearly absent in normal somatic cells yet crucial for tumor immortality, it remains a prime therapeutic target.

Telomerase inhibitors such as GRN163L (Imetelstat) induce telomere shortening and growth arrest in a variety of cancer models [3, 4]. However, resistance and inconsistent efficacy have also been reported, for example in pancreatic cancer cell lines where GRN163L-treated cells maintained very short but stable telomeres for extended periods before entering crisis [5]. These observations highlight the need for alternative approaches to disrupt telomere maintenance. One such approach is the use of G-quadruplex (G4) stabilizing ligands, which target guanine-rich telomeric DNA to block telomerase access and trigger DNA damage responses [6–8]. G4s are noncanonical secondary DNA structures formed in guanine-rich regions, including telomeres [9]. Stabilization of G4s with small molecules induces telomere dysfunction, providing a therapeutic avenue [10]. Our previous work established a quantitative assay for telomere-specific CIN using isogenic cell lines: one carrying a linear, telomere-containing EGFP-expressing HAC [11] and another with a circular, telomere-lacking HAC [12, 13]. This system distinguishes telomere-specific activity from off-target mitotic effects and validated G4 ligands as effective telomere-targeting agents [11, 14–17].

In addition to telomeric DNA, telomerase itself can be targeted at the protein level. HSP90 is a key chaperone required for hTERT folding, stability, and nuclear localization [18, 19]. Prior studies demonstrated that HSP90 inhibitors such as geldanamycin and 17-AAG destabilize hTERT, reducing telomerase activity [20–23]. Building on this, we tested a panel of HSP90 inhibitors (TAS-116, XL-888, SNX-2112, 17-AAG) for their ability to impair telomere maintenance. We further included GRN163L as a positive control. Here we show that these compounds disrupt telomerase function, induce telomere shortening, increase TIFs, and trigger CIN in human cancer cells, with TAS-116 exhibiting the strongest effects. These results underscore the therapeutic potential of HSP90 inhibitors, particularly TAS-116, as telomere-targeting anticancer agents.

## Methods

### Cell lines and culture

HT1080 cells carrying either a circular alphoidtetO-HAC-EGFP (lacking telomeres)[26] or a linear 21DqHAC-EGFP (containing telomeres) were cultured in Dulbecco’s modified Eagle’s medium (DMEM, Invitrogen) supplemented with 10% (v/v) fetal bovine serum (FBS, Clontech Laboratories, Inc.) and maintained under blasticidin S selection (4 μg/ml for circular HAC and 8 μg/ml for linear HAC). HEK293 cells carrying a GFP-21HAC2 were grown in the same medium supplemented with 10% FBS and maintained with 200μg/ml hygromycin B selection [25]. All cells were incubated at 37°C in a humidified 5% CO_2_ atmosphere. After initiation of drug treatment, cells were cultured in non-selective medium to allow detection of HAC loss. For experiments assessing mitotic abnormalities, parental HT1080 and HEK293 cells without HACs were cultured in DMEM supplemented with 10% FBS under non-selective conditions. Four HSP90 inhibitors—TAS-116, XL-888, SNX-2112, and 17-AAG—were tested. For CIN assays, drug concentrations were adjusted to the LC_50_ value of each compound in the respective cell line (Supplementary Table S1). For assays examining mitotic abnormalities, the LC_50_ value concentrations were used, as confirmed experimentally for HT1080 and HEK293 cells.

### Calculation of HAC loss and Flow cytometry

The rate of spontaneous HAC loss and HAC loss after HSP90 inhibitor treatment was calculated as previously described [12]. EGFP fluorescence expressions from HACs were measured by flow cytometry using a FACSCalibur instrument (BD Biosciences) with CellQuest software. Data was analyzed with FlowJo software. Cells were harvested by trypsinization, and at least 4 × 10^4^ cells were analyzed for each sample.

### Southern blot

Southern blot analysis of telomere length was performed using a dual 5′ biotin-labeled, telomere-specific probe. Genomic DNA (5 μg) was digested with HphI and MnlI restriction enzymes and separated on a 1% agarose gel by electrophoresis at 12 V for 20–24 h. DNA was transferred to a nylon membrane (Hybond-N+, GE Healthcare Life Sciences), denatured with an alkaline solution, and crosslinked by UV irradiation. The membrane was hybridized with ~ 200 pmol of a 20-bp telomere-specific probe (5′-CCCTAACCCTAACCCTAACC-3′; 5′ dual biotin-labeled) in ULTRAhyb^®^ ultrasensitive hybridization buffer (Invitrogen, AM8670) according to the manufacturer’s instructions. Detection was carried out using the Chemiluminescent Nucleic Acid Detection Module (Thermo Scientific, 89880).

### Quantitative PCR (qPCR) telomere length analysis

Telomere length was measured using the Absolute Human Telomere Length Quantification qPCR Assay Kit (ScienCell Research Laboratories, Carlsbad, CA, USA) according to the manufacturer’s instructions. Genomic DNA was extracted from drug-treated cells, and qPCR reactions were performed following the manufacturer’s protocol. Data analysis and calculation of telomere length were carried out using the standard curve method provided by the manufacturer.

### Immunofluorescence and micronuclei analysis

The number of γH2AX foci and the percentage of γH2AX foci colocalizing with telomeric sequences (identified by TRF2 staining) were quantified as previously described [11]. For each compound, approximately 20–30 nuclei were analyzed (Fig. 4 and Supplementary Table S5). After drug treatment, cells were fixed, permeabilized, and blocked as described in [11]. Samples were mounted with Vectashield Vibrance mounting medium containing DAPI. Images were acquired on a DeltaVision Core system (Applied Precision). Z-series were collected at 0.25 μm intervals, and image stacks were deconvolved using SoftWorx software.

Micronuclei (MNi) formation was assessed under the same experimental conditions. The presence of micronuclei was identified by DAPI staining and confirmed by fluorescence microscopy. The frequency of MNi formation was calculated as the percentage of nuclei containing at least one micronucleus.

### Telomere fluorescence in situ hybridization (Telo-FISH)

To obtain metaphase chromosome spreads, drug-treated cells were incubated overnight in growth medium containing 0.1 mg/ml Colcemid [11, 12, 35]. Metaphase cells were harvested, prepared, and hybridized with a TelC-PNA telomere probe (Cy3-OO-TAACCCTAACCCTAACCC; PNA Bio) [35]. Drug concentrations were the same as those used for Southern blot and qPCR assays, and GRN163L was included as a positive control (Supplementary Table S3). Slides were analyzed by fluorescence microscopy using the CRC/LRBGE Fluorescence Imaging Facility at NIH.

## Results

### HAC-based assay reveals telomere-specific effects of HSP90 inhibitors

**Figure 1:**
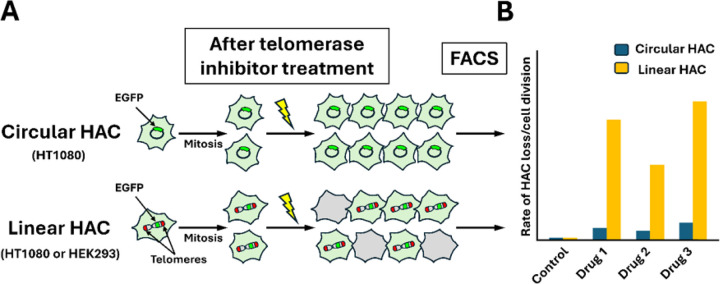
Experimental design of the HAC-based assay for measuring chromosomal instability (CIN) induced by telomere dysfunction. (A) Human HT1080 cells carried either the circular alphoid^tetO^-HAC (lacking telomeres) or the linear 21DqHAC (containing telomeres), while HEK293 cells carried the linear 21DqHAC. Both HACs stably segregate during normal cell division. Each HAC contains an EGFP transgene, allowing cells that inherit the HAC to display enhanced green fluorescence, whereas cells that lose the HAC lack fluorescence. In untreated control populations, cells maintain stable HACs and show uniform green fluorescence. In contrast, treatment with telomerase inhibitors leads to a greater loss of linear HACs compared to circular HACs, producing a mixed population of EGFP-positive and EGFP-negative cells. (B) The percentage of HAC-containing cells was quantified by flow cytometry (FACS). Compounds that promote HAC loss are thereby identified as inducers of chromosomal mis-segregation and CIN. Importantly, comparison of linear versus circular HACs allows telomere-specific effects of compounds to be distinguished from general cytotoxicity or mitotic defects.

To identify compounds that specifically target telomeres or telomerase, we used three cell lines each carrying a distinct HAC (Fig. 1). Two HT1080-derived lines carried either a linear, telomere-containing EGFP-expressing HAC (21DqHAC) [24, 25] or a circular, telomere-lacking alphoid^tetO^-HAC [26–28]. A third line, derived from HEK293, carried the same linear HAC. Linear HACs (~ 5 Mb) [11, 25, 29] contained functional kinetochores and segregated stably, while the circular HAC lacked telomeres but maintained mitotic stability [26–28]. EGFP expression enabled detection of HAC loss by flow cytometry (Fig. 1).

**Figure 2:**
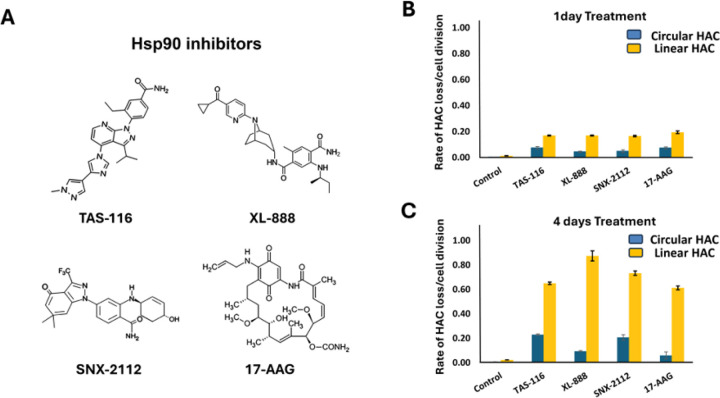
Effects of HSP90 inhibitors on the stability of circular versus linear HACs. (A) Chemical structures of the four HSP90 inhibitors used in this study. (B) Rates of HAC loss one day after treatment in HT1080 cells carrying either a circular or a linear HAC. (C) Rates of HAC loss four days after treatment under the same conditions. HT1080 cells containing either the circular alphoid^tetO^-HAC or the linear 21DqHAC were treated with HSP90 inhibitors at the concentrations described in the Supplementary Table S2. The rate of HAC loss was calculated based on the percentage of EGFP-positive versus EGFP-negative cells, as determined by flow cytometry. Untreated controls correspond to the frequency of spontaneous HAC loss. Compared with controls, all HSP90 inhibitors increased linear HAC loss, with TAS-116, XL-888, and SNX-2112 showing the strongest effects after 4 days of treatment, whereas the circular HAC remained largely stable.

Four HSP90 inhibitors were evaluated in this study: TAS-116, XL-888, SNX-2112, 17-AAG. (Fig. 2). LC50 values were determined for each compound in each cell line (Supplementary Table S1). HAC loss was measured after 1- and 4-day treatments (Fig. 2B and 2C). After 4 days, all HSP90 inhibitors increased linear HAC loss, whereas only mild effects were observed in circular HACs. As shown in Fig. 2C, fold increases in HAC loss relative to circular HACs were 6.8 (TAS-116), 8.3 (XL-888), 7.0 (SNX-2112), and 5.3 (17-AAG). One-day treatment did not produce significant effects. Comparable HAC loss was observed in both HT1080 and HEK293 cells carrying linear HACs, though HT1080 showed slightly higher sensitivity (Fig. 2 and Supplementary Figure S1). 17-AAG induced marked HAC loss in HT1080 cells but had weaker effects in HEK293, whereas TAS-116, XL-888, and SNX-2112 showed more consistent activity. GRN163L produced robust effects in both cell lines. (Supplementary Figure S1). The circular HAC remained stable under all treatments. These results validate the HAC-based assay as a tool for detecting telomere-targeting activity and identify TAS-116 as a potent inducer of telomere-specific CIN [30–32].

### HSP90 inhibitors induce telomere shortening in genomic chromosomes

**Figure 3:**
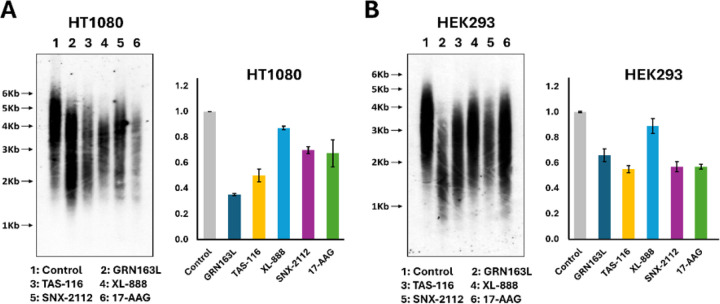
Effects of HSP90 inhibitors on telomere length in HT1080 and HEK293 cells. (A) Telomere length of HT1080 cells was assessed by Southern blot analysis and quantitative PCR after 20 days of treatment with HSP90 inhibitors. (B) Telomere length of HEK293 cells was evaluated under the same conditions. Different concentrations of each inhibitor were applied depending on the cell line, as described in the Supplementary Table S3. GRN163L, a telomerase inhibitor, was included as a positive control.

We next measured genomic telomere length in HT1080 and HEK293 cells by Southern blot and qPCR after 20 days of treatment with HSP90 inhibitors (TAS-116, XL-888, SNX-2112, 17-AAG) or GRN163L as positive control (Fig. 3). Concentrations were optimized for each line (Supplementary Table S3). TAS-116 induced the strongest shortening in both cell types (Fig. 3A, 3B). qPCR confirmed that TAS-116 reduced telomere length 1.8-fold in HT1080 and 2-fold in HEK293. GRN163L produced a 1.5-fold reduction in HT1080 and a 3-fold reduction in HEK293. Southern blot analysis supported these patterns, with statistical values provided in Supplementary Table S3B (HT1080) and S3C (HEK293). Collectively, TAS-116 emerged as the most effective HSP90 inhibitor for telomere shortening, with effects comparable to the positive control GRN163L.

### FISH confirms TAS-116–induced telomere dysfunction in HEK293 cells

To validate these findings, telomere signal intensity was quantified in HEK293 cells after 20 days of treatment with TAS-116, XL-888, or GRN163L (positive control), with untreated cells serving as a negative control (Supplementary Figure S2). Using FISH, positive control GRN163L reduced signal intensity to 55.15% of control (p < 0.0001). TAS-116 reduced signal intensity to 59.66% of control (p < 0.0001), while XL-888 reduced it to 70.78% (p < 0.0001) (Supplementary Figure S2B and Supplementary Table S4). These results corroborate the Southern blot and qPCR findings and confirm that TAS-116 is the most potent HSP90 inhibitor for inducing telomere dysfunction under these conditions.

### TAS-116 induces telomere-associated DNA damage and micronuclei formation

**Figure 4:**
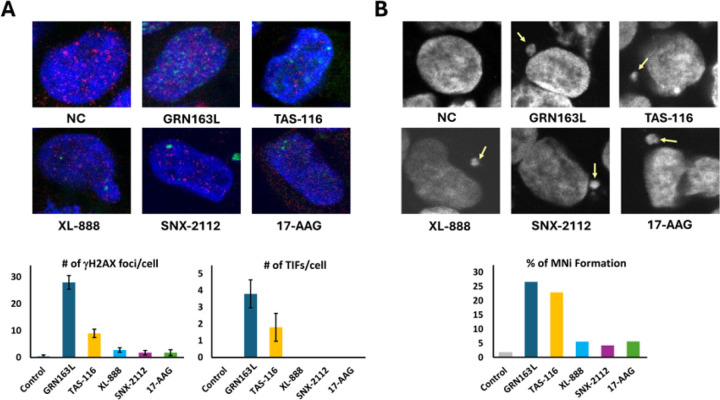
TAS-116 induces telomere-associated DNA damage and micronuclei formation in HEK293 cells. (A) Colocalization of γH2AX foci (green) with the telomeric protein TRF2 (red) in HEK293 cells treated for 20 days with TAS-116, XL-888, SNX-2112, 17-AAG, or the telomerase inhibitor GRN163L. Representative immunofluorescence images are shown (DNA counterstained with DAPI, blue). Colocalization of γH2AX and TRF2 signals indicates telomere dysfunction–induced foci (TIFs). Quantification of γH2AX foci and TIFs is presented in the accompanying graphs; at least 100 nuclei were analyzed per condition. (B) Micronuclei (MNi) formation in HEK293 cells after treatment with TAS-116, XL-888, SNX-2112, 17-AAG, or GRN163L. Representative images (arrows) illustrate MNi, and quantification is shown in the graph. TAS-116 and GRN163L induced significantly higher rates of MNi formation compared with other inhibitors and untreated controls. Statistical significance was determined by t-test.

To investigate the mechanisms of telomere dysfunction, we quantified DNA double-strand breaks (DSBs), telomere dysfunction–induced foci (TIFs), and micronuclei (MNi) in HEK293 cells treated with HSP90 inhibitors, with GRN163L included as a positive control (Fig. 4). γH2AX immunostaining revealed that GRN163L induced the highest number of DSBs (28 foci/cell, p < 0.0001), followed by TAS-116 (9 foci/cell, p < 0.0001). XL-888 (2.8 foci/cell), SNX-2112 (1.8 foci/cell), and 17-AAG (1.8 foci/cell) had weaker effects (Fig. 4A and Supplementary Table S5A).

TIFs, defined as colocalization of γH2AX with the telomeric protein TRF2, showed that TAS-116 induced on average 1.8 TIFs per TIF-positive nucleus, whereas GRN163L induced 3.8, and no TIFs were detected with the other inhibitors (Fig. 4A and Supplementary Table S5A).

MNi formation, a marker of CIN, was highest with GRN163L (26.62%, p < 0.0001) and TAS-116 (22.98%, p < 0.0001). Lower rates were observed with XL-888 (5.61%), SNX-2112 (4.31%), and 17-AAG (5.66%) compared to 1.79% in controls (Fig. 4B and Supplementary Table S5B). Representative images are shown in Fig. 4B.

These findings, consistent with prior assays, confirm that TAS-116 and GRN163L induce strong telomere-specific DNA damage and CIN, with TAS-116 emerging as the most potent HSP90 inhibitor in this context.

## Discussion

The rigorous evaluation of telomere-targeting agents is critical for advancing cancer therapeutics, yet few assays can reliably distinguish telomere-specific activity from general cytotoxicity. By employing an HAC-based system with linear and circular HACs, combined with direct analyses of telomere length and function, we established a versatile framework for identifying compounds that disrupt telomere integrity.

Our findings demonstrate that TAS-116, an HSP90 inhibitor currently in clinical development, exhibits robust telomere-targeting effects across multiple assays. TAS-116 induced linear HAC loss, genomic telomere shortening, reduced telomere signal intensity, elevated TIFs, and increased micronuclei formation. These effects were comparable in magnitude to those of the positive control GRN163L, a direct telomerase inhibitor, confirming the validity of our assay. All HSP90 inhibitors tested promoted telomere dysfunction to varying degrees, underscoring that HSP90 inhibition broadly impacts telomere integrity, with TAS-116 showing the strongest and most reproducible effects across assays.

**Figure 5:**
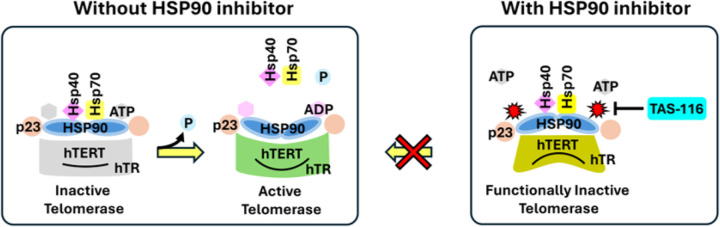
Model of telomerase regulation by HSP90 and its inhibition by TAS-116. Schematic representation of the role of HSP90 in telomerase folding and activation, and how TAS-116 disrupts this process. Under normal conditions (right panels), hTERT is associated with HSP90 and co-chaperones such as p23 in an ATP-dependent manner. ATP binding and hydrolysis drive the HSP90 chaperone cycle, allowing proper folding, stabilization, and assembly of hTERT into an active telomerase holoenzyme with hTR and associated factors. This results in a functionally active telomerase complex capable of elongating telomeres and maintaining chromosomal stability. In the presence of TAS-116 (left panel), the inhibitor occupies the ATP-binding pocket of HSP90, blocking its ATPase activity and halting the chaperone cycle. Consequently, hTERT fails to achieve correct folding and assembly, leaving telomerase functionally inactive. This inhibition of telomerase activity contributes to telomere dysfunction and chromosomal instability (CIN), providing a mechanistic basis for TAS-116’s anticancer effects.

Mechanistically, TAS-116 binds to the ATP-binding pocket of HSP90 and arrests its chaperone cycle, thereby impairing proper folding, stabilization, and assembly of hTERT (Fig. 5). Consequently, telomerase is rendered functionally inactive, consistent with defective holoenzyme assembly and impaired telomere elongation. This explains how TAS-116 indirectly suppresses telomere maintenance while simultaneously destabilizing other oncogenic client proteins of HSP90.

Importantly, GRN163L was included as a positive control because our previous work had established its strong activity as a telomerase inhibitor capable of inducing telomere dysfunction when tested in the same HAC-based system. Its reproducible activity across both linear and circular HAC assays provides a reliable benchmark against which novel compounds can be evaluated. As expected, GRN163L consistently triggered robust telomere shortening, DNA damage, and CIN, thereby validating the sensitivity of our experimental framework.

Beyond these results, our study contributes to the broader understanding of telomere-directed therapies. Cancer cells rely heavily on telomere maintenance for survival, and strategies such as telomerase inhibition, G-quadruplex stabilization, and Shelterin disruption have all been pursued with varying success. HSP90 inhibition offers an alternative route, indirectly destabilizing telomerase through impaired hTERT folding and assembly while simultaneously impairing multiple oncogenic pathways. The dual functionality of TAS-116—chaperone inhibition and telomere destabilization—may enhance its therapeutic potential by exploiting a fundamental vulnerability of cancer cells. In line with this, TAS-116 has undergone evaluation in a first-in-human phase I clinical trial for solid tumors, supporting the translational relevance of its telomere-targeting activity [33, 34].

This work also underscores the methodological value of HAC-based assay. By directly comparing linear and circular HACs, this approach distinguishes telomere-dependent from off-target effects, providing clarity that traditional assays cannot achieve. Such precision makes this system a powerful tool for preclinical evaluation of telomere-targeting agents.

Limitations of this study include its reliance on in vitro models and a relatively short treatment window, which may not fully capture long-term telomere erosion or the complexity of *in vivo* tumor biology. Nevertheless, these limitations point to clear next steps: extending analyses to animal models and expanding compound screening. Importantly, they do not diminish the strong and consistent evidence supporting TAS-116 as a telomere-targeting agent.

In conclusion, our results establish TAS-116 as a potent telomere-targeting compound. They also validate the HAC-based assay as an effective framework for evaluating telomere-directed therapeutics. As illustrated in Fig. 5, HSP90 normally supports telomerase activity by stabilizing and folding hTERT in an ATP-dependent manner, enabling assembly of an active holoenzyme. TAS-116 blocks this cycle by occupying the ATP-binding pocket of HSP90, thereby rendering telomerase functionally inactive. Together, these findings highlight the clinical promise of TAS-116 while underscoring the broader utility of HAC-based and telomere assays in guiding the development of novel cancer therapies.

## Conclusions

TAS-116 consistently disrupts telomere maintenance—inducing linear HAC loss, telomere shortening, reduced telomere FISH signal, elevated TIF formation, and increased micronuclei—thereby validating the HAC-based system as a robust platform for detecting telomere-directed activity. Collectively, these findings highlight the therapeutic potential of HSP90 inhibition as an effective strategy to target telomere maintenance in cancer cells.

## Supplementary Material

Supplementary Files

This is a list of supplementary files associated with this preprint. Click to download.

- SupplementaryFiles.docx

## Data Availability

All data used and/or analyzed during this study are available from the corresponding author on reasonable request.

## Abbreviations

CIN: Chromosome Instability
HSP90: Heat Shock Protein 90
HAC: Human Artificial Chromosome
FISH: Fluorescence in situ Hybridization
TIF: Telomere dysfunction-Induced Foci
MNi: Micronuclei
EGFP: Enhanced Green Fluorescent Protein
TRF2: Telomeric Repeat-binding Factor 2
